# GproteinDb in 2024: new G protein-GPCR couplings, AlphaFold2-multimer models and interface interactions

**DOI:** 10.1093/nar/gkad1089

**Published:** 2023-11-24

**Authors:** Gáspár Pándy-Szekeres, Luis P Taracena Herrera, Jimmy Caroli, Ali A Kermani, Yashraj Kulkarni, György M Keserű, David E Gloriam

**Affiliations:** Department of Drug Design and Pharmacology, University of Copenhagen, 2100 Copenhagen, Denmark; Medicinal Chemistry Research Group, HUN-REN Research Center for Natural Sciences, Budapest H-1117, Hungary; Department of Drug Design and Pharmacology, University of Copenhagen, 2100 Copenhagen, Denmark; Department of Drug Design and Pharmacology, University of Copenhagen, 2100 Copenhagen, Denmark; Department of Structural Biology, St. Jude Children's Research Hospital, Memphis, TN 38105, USA; Department of Drug Design and Pharmacology, University of Copenhagen, 2100 Copenhagen, Denmark; Medicinal Chemistry Research Group, HUN-REN Research Center for Natural Sciences, Budapest H-1117, Hungary; Department of Drug Design and Pharmacology, University of Copenhagen, 2100 Copenhagen, Denmark

## Abstract

G proteins are the major signal proteins of ∼800 receptors for medicines, hormones, neurotransmitters, tastants and odorants. GproteinDb offers integrated genomic, structural, and pharmacological data and tools for analysis, visualization and experiment design. Here, we present the first major update of GproteinDb greatly expanding its coupling data and structural templates, adding AlphaFold2 structure models of GPCR–G protein complexes and advancing the interactive analysis tools for their interfaces underlying coupling selectivity. We present insights on coupling agreement across datasets and parameters, including constitutive activity, agonist-induced activity and kinetics. GproteinDb is accessible at https://gproteindb.org.

## Graphical Abstract

**Graphical Abstract:**
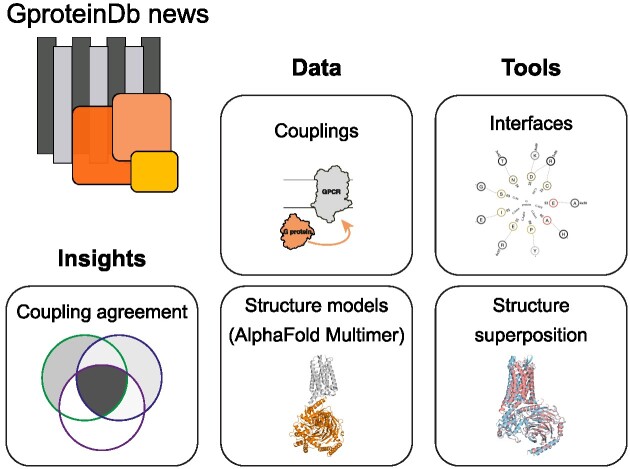

## Introduction

G proteins are the major signal proteins of ∼800 receptors for hormones, neurotransmitters, tastants and odorants, including numerous drug targets (1–3). There are 16 human G proteins grouped in four families with distinct downstream signaling pathways: G_s_ (G_s_ and G_olf_), G_i/o_ (G_i1_, G_i2_, G_i3_, G_o_, G_z_, G_t1_, G_t2_, G_gust_), G_q/11_ (G_q_, G_11_, G_14_ and G_15_) and G_12/13_ (G_12_ and G_13_). Despite their relatively low numbers, the G protein subtypes/families can mediate numerous distinct cellular and physiological responses as receptors selectively engage different G protein profiles spatially and temporally (tissue and kinetic selectivity, respectively) (4–6). Furthermore, the same receptor, when bound to different ligands, can exhibit biased signaling via alternative G proteins or other signaling proteins—a mechanism allowing for design of safer drugs by avoiding undesired G protein signaling causing adverse effects (7–9). Hence, it is of great importance to advance fundamental structure-function of G protein signaling and to explore new therapeutic mechanisms, including biased ligands and inhibitors of constitutively active cancer mutants (10).

GproteinDb is an online research platform first published in 2022 (11). In addition to a dedicated web site (https://gproteindb.org), its menu system is also available via GPCRdb (2), ArrestinDb (12) and Biased Signaling Atlas (13) covering related signal transduction proteins and research communities. The first version established e.g. an atlas of G protein couplings integrating three major datasets (6), crystal and cryo-EM structures with annotations extending PDB (14), tailored tools to investigate GPCR–G protein interface interactions and predicted selectivity determinants enabling mutagenesis experiments. Despite being relatively new, GproteinDb has already supported a breadth of original research studies spanning e.g. autoregulation of signaling (15), allosteric modulation (16)/binding sites (17), GPCR–G protein selectivity (18) as well as community guidelines for ligand bias (19) and a review on GPCR signaling (20).

**Figure 1.**
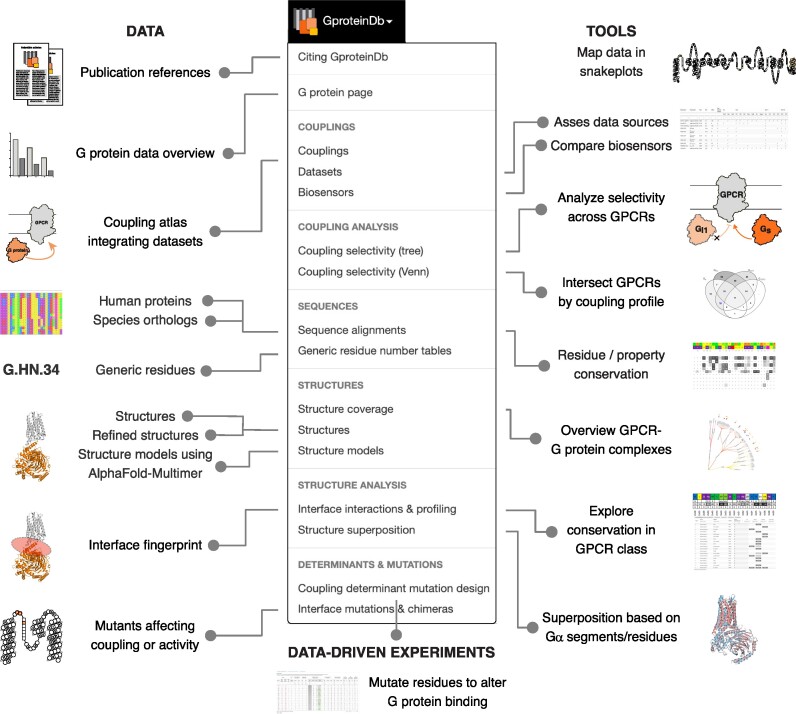
G protein data, analysis resources and data-driven experiment tools in the GproteinDb 2024 release.

Here, we present the first major update of GproteinDb greatly expanding its structural templates, adding AlphaFold2 structure models of GPCR–G protein complexes and advancing the interactive analysis tools for their interfaces underlying coupling selectivity. These data and tools should serve to extend the use cases of this comprehensive research platform (Figure 1) across computational, structural, pharmacological and drug design applications.

## Methods

### G protein coupling data processing

This release excludes data from chimeric G proteins. For this reason, we only include G_q_ (wildtype) couplings from the Inoue TGFα shedding dataset (5) and chose not to include a new dataset from the Orlandi lab (21). For the Inoue TGFα shedding dataset we also applied a cutoff log(Emax/EC_50_) ≥ 7.0, which were based on the largest agreement with couplings and non-couplings determined by both the Bouvier GEMTA and Martemyanov FreeGβγ-Nluc datasets. The new Lambert RGB-GDP datasets (22–24) were subjected to a cutoff of 0.009 (defined by outlier identification with a false discovery rate of 1%). The GEMTA biosensors label/measure distinct effectors of the G_i/o_, G_q/11_ and G_12/13_ families and EMTA (G_s_) labels G_α_ (4). To enable comparison across G protein families, we min-max normalized Emax values from each of these four biosensors setting the highest value among all GPCR – G protein pairs to 100. No normalization was needed for the other biosensors for which the different G protein subtypes are measured with the same labeled molecules. For the two 5-HT_7_ receptor isoforms in the Martemyanov FreeGβγ-Nluc dataset (25), we used the full-length isoform, 5-HT_7A_ while excluding the truncated isoform, 5-HT_7B_. Furthermore, the heterodimer of GABA_B1_/GABA_B2_ was stored for GABA_B2_, as GproteinDb does not yet capture receptor dimers. The MS Excel file containing all coupling datasets and their processing for import into GproteinDb can be downloaded via a link in the *Couplings* page.

### Refining G protein crystal and cryo-EM structures

We updated our refined complex structures from (11) to use our AlphaFold2-Multimer complex models (next section). Hence, instead of using experimental structures of other receptors, the AlphaFold2-Multimer model of the same receptor is used as swap-in template to fill in missing coordinates of Gα proteins and receptors. Each structure and swap-in template were superposed based on the backbones of four residues flanking both sides (one side for termini) of the segment missing coordinates. To avoid clashes, GPCR C-termini that were swapped in from the AlphaFold model were truncated to 10 residues. Finally, any residues with clashes or flanking swap-in sites were optimized using MODELLER (26).

Mutated residues were reverted to wildtype. For chimeric Gα proteins, we used the subtype described by authors when this referred to a single subtype. When authors listed multiple wildtype Gα subunits, we used the construct sequence of G.H5 to determine the subtype. We mapped the residues of chimeric Gα constructs to wildtype sequences by implementing a BLAST-like sequence alignment. This resolved incorrect sequence mappings and increased the quality of structure refinement of the G proteins. The alignment of chimeras and wildtype Gα subunits can be found in FASTA format at https://github.com/protwis/gpcrdb_data/blob/master/g_protein_data/g_protein_chimeras_gapped.fasta.

### Building GPCR–G protein complex structure models

We build models of GPCRs with the heterotrimeric G proteins that have been identified as the primary transducer from one of the quantitative coupling datasets in GproteinDb (1124 such models completed at time of writing). Furthermore, we build complex models of every human G_α_ protein and human GPCR in GPCRdb (2). All models were built using AlphaFold2 version 2.3.1 with the Multimer option and amber relaxation (27). Structural templates were included up to 1 January 2023. We employed a diverse set of metrics to assess model quality. Firstly, to evaluate the overall structural quality, we employed AlphaFold2’s predicted version of the template modeling (TM) score. The TM-score is calculated by measuring pairwise distances between a superimposed predicted model to a reference structure. It refines itself through various superimpositions, reducing the influence of local variations and emphasizing topological similarity. The predicted version provided by AlphaFold2, known as pTM, is an estimate of the TM-score, that uses a neural network to predict the Cα positioning errors for TM score calculations. Secondly, we used the interface predicted TM-score (ipTM) to assess the quality of the interface between the GPCR and the Gα. The interfaces are evaluated by comparing residue interactions between protein chains. We established thresholds for pTM ≥0.5 and ipTM ≥0.2, both ranging from 0 to 1, with 1 signifying a highly accurate model. This choice was guided by our examination of the relative positioning of the Gα and GPCR, with emphasis on preventing loop knots. A pTM cutoff of ≥0.5 indicated good overall model quality and enabled us to adopt a more permissive ipTM (≥0.2). This approach ensured the expected positioning of the Gα relative to the GPCR. These cut-offs are consistent with the levels of significance on the TM scores identified in previous studies assessing protein fold similarity (28). Thirdly, we also assessed the relative positioning of Gα and GPCR using the predicted aligned error (PAE). The PAE is a matrix that represents the positional error of the Cα of predicted models (29). Utilizing the data from this matrix, we calculated the PAE mean focusing on the section that scores the position of the Gα in respect to the GPCR. We excluded long flexible C and N termini of the GPCRs due to the uncertainty they introduce. Furthermore, with PAE scores ranging from 0 to 31.75 Å, we established a cut-off of 26 Å for the PAE mean score of compared residues (even if the ipTM score was satisfactory). Finally, we visualized the local per-residue consistency within models using predicted local Distance Difference Test (plDDT) values (30) which span 0–100.

To increase model accuracy and avoid cross-chain clashes, it was necessary to truncate or delete very long receptor segments in some GPCR classes. For Class A GPCRs, the third intracellular loop 3 was shortened to 14 residues. For Class B2 (*Adhesion*) GPCRs, we truncated 18 receptors directly after their GPS site (HL|T/S motif) in line with their self-activating mechanism where GPS cleavage causes the truncated N-terminal ‘Stachel’ sequence to activate the seven-transmembrane domain (31). Except for AGRA1 which has a short N-terminus, the N-termini of the remaining 14 class B2 receptors lacking a GPS site were also truncated just before the first transmembrane helix—as were Class C GPCRs (removing the large ligand-binding Venus Flytrap domain (VFTD)).

### Structure superposition tool

A Structure superposition tool was developed by modifying and extending the code for a previously published similar tool for GPCRs (32) and implementing the Common Gα numbering of residues (33).

### GPCR–G protein interface interactions

We expanded the interface interaction tools (*Interface interaction & profiling* page) to include our refined complex structures and new complex models. To enable detailed investigation of specific complexes, we developed a ‘biflare plot, a visualization tool for interface interactions in single complexes. It uses a customized logic for spatial amino acid placement. Gα residues are placed into an inner circle and are ordered based on segment and generic residue number. GPCR residues are positioned in an outer circle based on the average angle calculated across all the interacting Gα residues and the center of the inner circle. Javascript and the D3 library were used to design and develop the visualization of the plot. Python and the Django framework were used for data handling and processing.

### Application Programming Interface (API)

We used the REST framework (https://www.django-rest-framework.org/) to implemented API calls enabling programmatic retrieval of G protein data.

## Results

### New coupling data and *Datasets* page

GproteinDb serves as a one-stop-shop for G protein couplings integrated from multiple datasets which can now be browsed in a new page *Datasets*. This release has added three new datasets (lab and biosensor): Martemyanov FreeGβγ-Nluc (25), Lambert RGB-GDP (22–24) and Inoue NanoBiT-G (5), respectively. While the new datasets cover fewer G protein subtypes (4–8 compared to 12–15) they represent all four G protein families. However, G_15_ couplings to GPCRs often differ substantially from other members of the G_q/11_ family (4,6,25) and were not tested in the Inoue NanoBiT-G and 2019–2020 Lambert RGB-GDP publications. The Inoue NanoBiT-G couplings, like the previously included, represent log(Emax/EC_50_) values (6,11). In contrast, the Martemyanov FreeGβγ-Nluc (25) and Lambert RGB-GDP (22–24) datasets bring the new parameters activation rate (s^−1^) and *E*_constitutive_ (efficacy from constitutively active receptors), respectively. Interestingly, the latter dataset spans 48 orphan GPCRs not activated by an agonist ligand (24) and 27 other, non-orphan receptors both with and without ligand. Measuring constitutive activity made it possible to profile the couplings of 22 of the 48 orphan receptors that lack agonists and are therefore not covered by previous datasets, which typically use the endogenous ligand. The increased coverage of couplings from other datasets, and a more stringent view on the biological system, led us to remove data for chimeric G proteins—reducing Inoue TGF-α shedding dataset to only G_q_, which is wildtype.

### New *Biosensors* page

A new page *Biosensors* provides an overview of the biosensors used to determine the couplings deposited in GproteinDb (4–5,22,24,34–42). To make it easier to track the evolution of biosensors, we introduce a classification of biosensors by their type, which is defined based on the labeled molecules (column Biosensor type). For example, the biosensor type GG refers to G_α_–G_βγ_ dissociation assays that arose in the mid 2000s (GABY) (35) and have been optimized and expanded to additional reporters and G protein subtypes in 2019 (NanoBiT-G) (5) and 2020 (TRUPATH) (37), respectively. To clarify what has been measured we list the measured process (e.g. G_α_–G_βγ_ dissociation) and its downstream steps (after receptor binding). For further reference, we provide the original publication year and link.

### New *Couplings* page

This release features a new *Couplings* page with unique layout and functionality. A new section of the data browser tabulates the number of supporting datasets for G protein family couplings to a given GPCR. This allows filtering all couplings (default) to a subset with experimental evidence from several sources. To show G protein coupling selectivity or promiscuity of GPCRs, we provide: (i) a G protein family count, (ii) the primary transducer family/subtype (marked 1′), (iii) family rank orders (1′, 2′, 3′ and 4′) and (iv) family/subtype percent activity (relative primary transducer). This allows swift cross-dataset analysis of any GPCR’s coupling profile or a primary transducer's set of receptors. Furthermore, the family rank orders contain the note ‘nc’ for experimentally determined non-couplings—enabling analyses that contrast sets of coupling versus non-coupling GPCR–G protein pairs, for example to discover determinants of coupling selectivity.

Each GPCR has multiple rows – one for each dataset. However, non-redundantconsensus couplings can be shown by applying a filter “GproteinDb” in the “Lab” column. The GproteinDb row contains a mean across datasets in the family/subtype percent activity sections and use these to calculate consensus family rank orders (for receptors with only GtoPdb data this is just 1’ or 2’). This release presents alternative coupling parameters E_constitutive_ (efficacy from constitutively active receptors), log(Emax/EC_50_), and activation rate (s^−1^), which are defined first in the section ‘Quantitative values’. Selected couplings (via filtering) can be downloaded as a MS Excel spreadsheet for further analysis. Furthermore, the *Couplings* page provides a link to the MS Excel file via which all couplings were imported to GproteinDb and containing additional raw data, formulas (for normalizations, cut-offs etc.) and statistical significances (SDs or SEMs) not shown online.

### Updated Coupling selectivity (tree)/(Venn) pages

The *Coupling selectivity (tree)* page maps the G protein family couplings onto trees of receptors divided by class (separate trees) and classified by their ligand types and receptor families (share endogenous ligand) (11). The *Coupling selectivity (Venn)* page intersects G protein families and provides a list of receptors based on their coupling selectivity. To use GproteinDb's new coupling datasets, both pages now include all couplings datasets and an option to use all couplings or only those supported by the Guide to Pharmacology (GtoPdb) database or two or more other datasets.

### Structures and refined structures

The *Structures* page is updated quarterly with new structures from the Protein Data Bank (structures from RCSB (43) and SIFTs data from PDBe (14)) enriched with annotation of the overall structure (publication date, experimental method, resolution), receptor (name, chain ID), ligand (name, type, PubChem ID, modality), nanobodies, fusion proteins, G proteins (name and chain IDs of subunits), arrestins (name, chain ID), RAMPs and GRKs. The associated *Structure coverage* page overviews the availability of structures across G protein families, GPCRs and GPCR classes. There are currently 690 G protein structures, whereof 544 are structures of GPCR - G protein complexes. The structures are also available in a refined version which models-in missing segments (truncated/deleted from the construct sequence or not resolved in density map) and reverts mutated residues to wildtype. GproteinDb also provides a refined sequence of complex structures. Specifically, the residue numbering in chimeric G_α_ subunits is mapped to wildtype sequence and gets assigned generic residue numbers with the common G protein residue numbering scheme (44).

### Structure models

**Figure 2.**
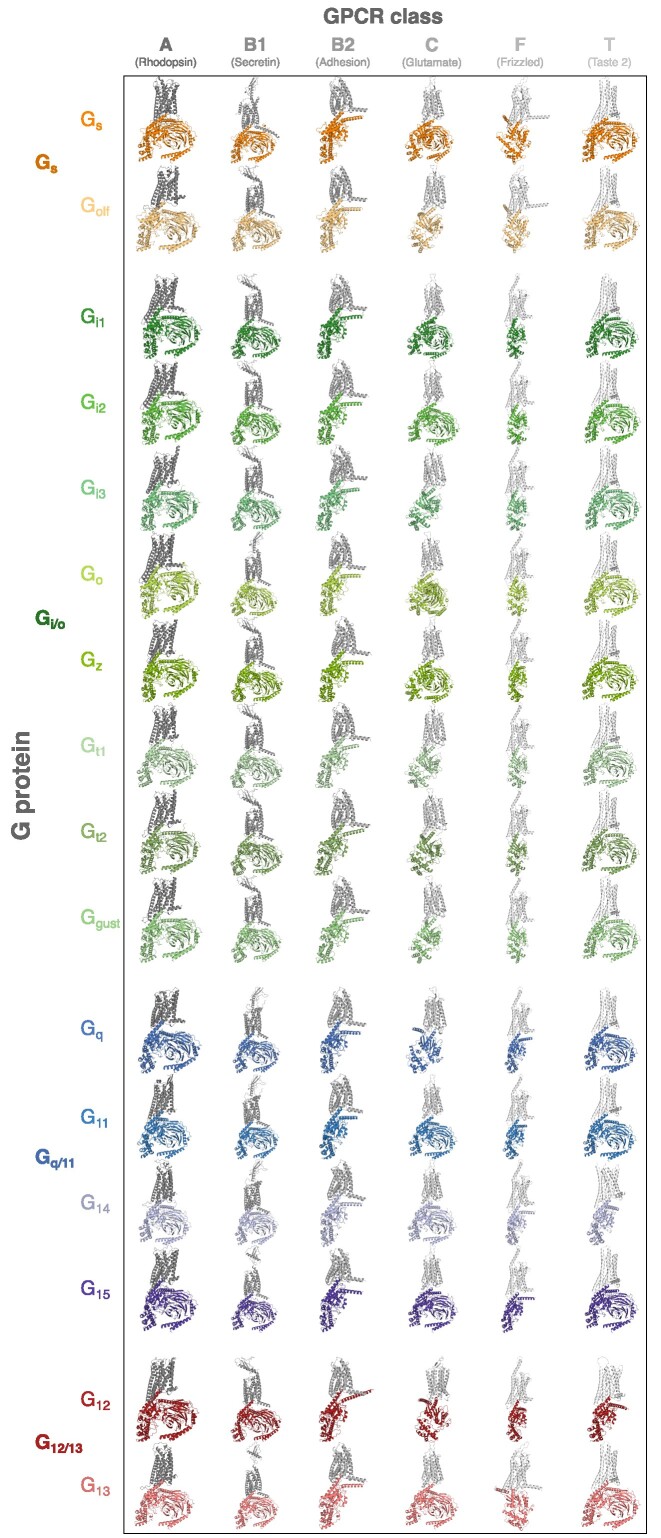
GproteinDb features AlphaFold2-Multimer-based (27) structure models of GPCRs and the heterotrimeric G protein (Gαβγ complex) that constitute its primary transducer G protein. Furthermore, GproteinDb builds models of the theoretical interactome spanning all 16 human Gα subunits and 425 human GPCRs in GPCRdb (2). The figure shows representative such models whereof the following complexes are with heterotrimeric G proteins (Class A – all, Class B1 – all, Class C – all except G_olf_, G_i3_, G_t1_, G_t2_, G_gust_, G_12_ and Class T – all except G_14_, G_12_) and the remaining are Gα–GPCR complexes.

The *Structure models* page presents models of human G protein–GPCR complexes. The first publication of GproteinDb contained homology models of GPCR–G protein complexes built using MODELLER (26). However, these models were limited in numbers and quality due to the relatively few complex templates at the time (see Discussion). This release features AlphaFold2-Multimer-based (27) structure models of 1121 GPCRs and heterotrimeric G proteins, including all primary transducers. Furthermore, we present 5595 models of the 16 human Gα subunits and 425 human GPCRs in GPCRdb (2) spanning the GPCR classes (families) A (*Rhodopsin*), B1 (*Secretin*), B2 (*Adhesion*), C (*Glutamate*), F (*Frizzled*) and T (*Taste 2*) (Figure 2). This represents 82% of all theoretical G protein–GPCR complexes and the remainder will be added in coming releases as they pass our quality filters (see Methods). Whereas not all complexes will occur biologically, it is difficult to definitely rule out unseen couplings as they depend on many factors e.g. ligand and tissue/cell type and having access to the models may still be useful to inform studies investigated for example GPCR–G protein selectivity (33,45). To allow users to select models of only validated complexes, the *Structure models* page features sorting and filtering based on GproteinDb's experimental coupling data.

We provide several model quality metrics. The 3D viewer colors residue by pLDDT scores, which assess the local per-residue consistency within models using the predicted local Distance Difference Test (30). Furthermore, the quality is visualized in horizontal bars placing the model score within the respective range limits of three scores; pTM (overall model), ipTM (protein interface) and PAE mean (positioning of Gα relative GPCR) (30). Together, this comprehensive system grants users a swift, yet in-depth perspective on the quality and reliability of the G protein-GPCR complex models.

### New structure superposition tool

We present a new tool to superposition G protein–GPCR complex structures or models in GproteinDb. This tool is available from a dedicated page *Structure superposition* as well as within the pages *Structures* and *Structure models* (via the ‘Superposition’ button). The tool works by first selecting a reference structure and then additional structures/models for superposition—based on a custom set of Gα segments or residues (defined based on the Common Gα numbering (CGN) scheme (33)). Finally, the superposed structures/models are downloaded (in PDB format) for visualization in any software.

### G protein–GPCR interface interactions in a single structure

**Figure 3.**
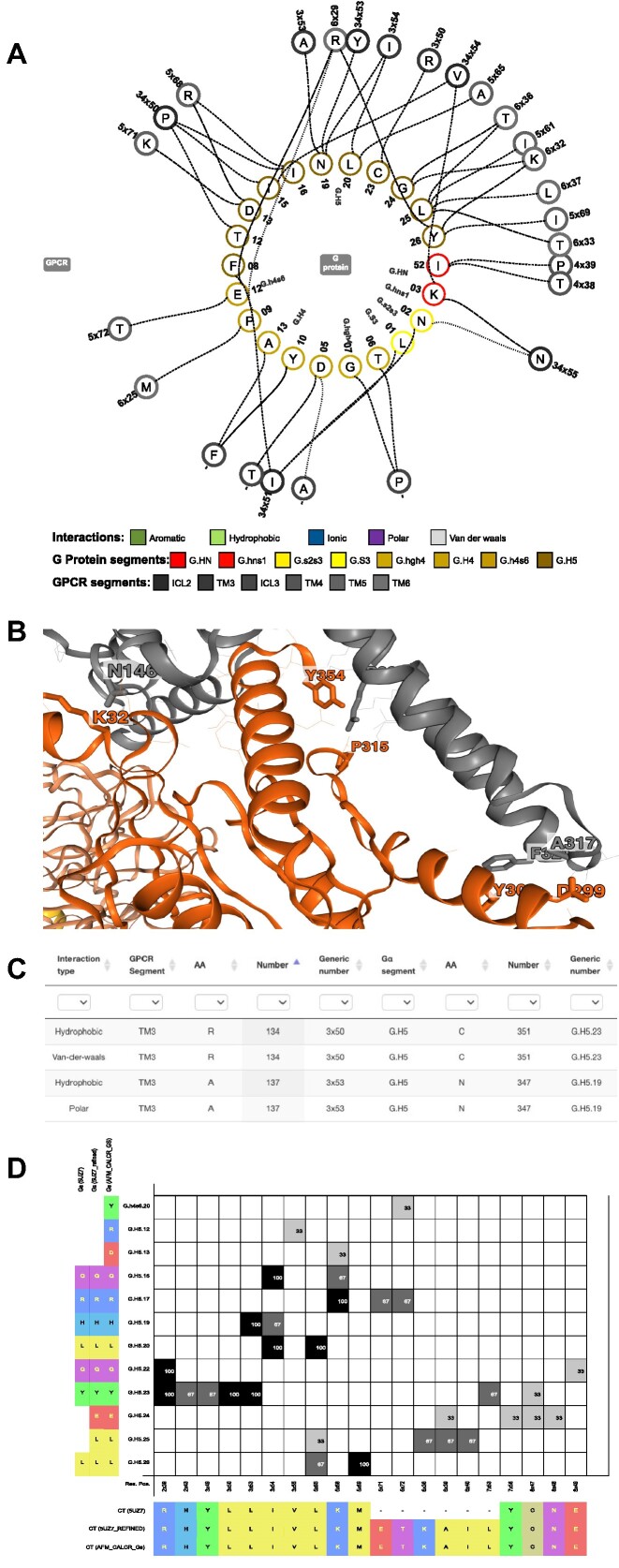
G protein-GPCR interface interactions in a single (A–C) or multiple (D) structure(s). (**A**) Biflare plot showing all residue-residue interactions in G_α_–GPCR complex (here G_o_–5HT_1A_ model). (**B**) 3D view labeling interacting residues with amino acid number (G_α_ orange, GPCR red). Residues are depicted with sticks and line representation denoting interactions with strict and loose definitions, respectively (from (46), defined in a tool tip). (**C**) Interaction browser featuring sorting and filtering of interactions based on sequence, generic residues numbers, segments and interaction types. (**D**) GPCR–G protein interface interaction matrix illustrating expanded data from refined structures and models. The matrix shows GPCR–G protein residue interactions for the same complex (G_s_–Calcitonin (CT) receptor) across the unrefined structure (PDB: 5UZ7) (54), GproteinDb's refined structure modeling-in missing residues/atoms (5UZ7_refined) and AlphaFold2-Multimer model (AFM_CALCR_Gs) (54). Of note, these display an increasing number of interface interactions; 14, 20 and 26, respectively.

The *Structures* and *Structure models* pages have data browsers that link on to a dedicated page for the selected structure, refined structure or model. In this release, this page has been equipped with four visualizations of interface interactions (Figure 3). Firstly, a ‘biflare’ plot shows two concentric circles for G protein and GPCR residues, respectively connected by differently dashed lines indicating their type of molecular interaction (Figure 3A). This plot can show many or fewer interactions based on strict and loose definitions, respectively (from (46), defined in a tool tip) and toggle between backbone/sidechain interactions or a single selected G protein or GPCR segment or residue. Furthermore, the biflare plot offers color-coding by interaction type, segments or amino acid properties. Secondly, a 3D viewer shows the structure, refined structure or model as an interactive cartoon representation where the interacting residues are labeled with their one-letter amino acid code and sequence number (Figure 3B). Interacting residues are depicted with sticks and line representation denoting interactions with strict and loose definitions, respectively. Thirdly, an interaction data browser offers sorting and filtering of interacting residues by generic residue number and interaction type (Figure 3C). These data can also be downloaded (MS Excel spreadsheet) enabling analysis in other software. Finally, a matrix (similar to Figure 3D) shows interacting Gα and GPCR residues (Y- and X-axes, respectively). Interactions (matrix cells) are colored by interaction type and amino acids are colored by property.

### G protein–GPCR interface interactions across several structures

The *Interface interactions and profiling* page features interactive analysis tools and visualizations of residue-residue interactions across several GPCR–G protein interfaces. The application of these tools has here been greatly expanded through the addition of our new structure models increasing the number of GPCR–G protein complexes that can be analyzed/visualized from 527 to >6000. Furthermore, as many experimental structure complexes have poor density (330 out of 527 structures have a resolution worse than 3.0 Å) their interface residues are often missing sidechain atoms leading to lacking interactions. This is addressed in this update by adding the option to analyze refined structure complexes and AlphaFold2 complex models (Figure 3D). This enables investigation of residue interactions with increased completeness on the residue and protein complex levels by combining any type of structural templates (*Type* column under the *Structure* section). Furthermore, it makes it possible to assess how Gα chimera or mini-G proteins (47) affects GPCR coupling and residue-level interactions.

### Application Programming Interface (API)

G protein-related data can be programmatically accessed through our API at https://gproteindb.org/services.

## Discussion

**Figure 4.**
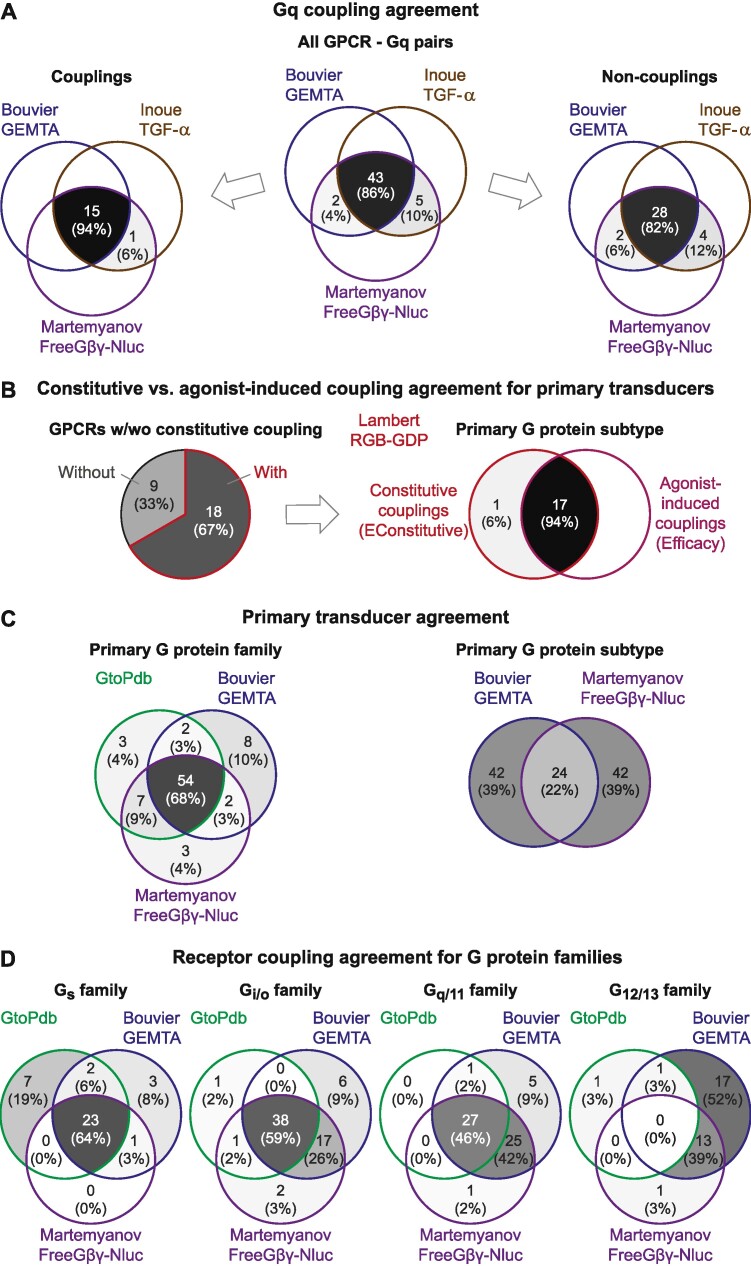
Agreement of couplings across datasets and parameters. (**A**) Intersection of Gq couplings for 50 shared GPCRs in the Bouvier GEMTA, Inoue TGF-α shedding and Martemyanov FreeGβγ-Nluc datasets. The large agreement was achieved by filtering (only) Inoue couplings requiring log(Emax/EC_50_) ≥ 7.0 to account for downstream signal amplification. Couplings and non-couplings were defined based on support from at least two out of three datasets (majority rule). (**B**) Intersection of constitutive and agonist-induced couplings from the Lambert RGB-GDP datasets. (**C**) Intersection of the primary transducers of 66 shared GPCRs (79 combinations) in the GtoPdb, Bouvier GEMTA and Martemyanov FreeGβγ-Nluc datasets. (**D**) Intersection of the receptor sets among, the 66 shared GPCRs, coupling to each G protein family from the GtoPdb, Bouvier GEMTA and Martemyanov FreeGβγ-Nluc datasets. (A–D) The underlying datasets and calculations of above agreements are available in the file GPCR-G_protein_couplings.xlsx available from the *Couplings* page. Grey-scaling, numbers and percentages were obtained from VENNY 2.1 (https://bioinfogp.cnb.csic.es/tools/venny/index.html).

The new *Couplings* page greatly facilitates exploration of GPCR – G protein selectivity by allowing filtering based on primary transducer, rank orders and percent activities. Importantly, these descriptors enable comparisons across all datasets, and replace the previous common parameter mean log(Emax/EC_50_) across datasets, which may not be meaningful as the underlying biosensors differ substantially. For example, the biosensors measure different molecules and processes at downstream signaling steps ranging; 0: RGB and RGB-GDP, 1: GABY, TRUPATH and NanoBiT-G, 2: FreeGβγ-Nluc and EMTA, 3: GEMTA and 5: TGFα-shedding (see *Biosensors* page). To account for excessive signal amplification from the TGF-α shedding biosensor, we applied a filter requiring log(Emax/EC_50_ ≥ 7.0). Notably, this achieves an agreement of 94% for couplings and 82% for non-couplings between Gq and GPCRs compared to the EMTA/GEMTA and FreeGβγ-Nluc datasets (Figure 4A). Our *Biosensors* page introduces a classification of biosensor types facilitating comparison and tracking of their evolution to improve signal-to-noise ratios and utility. Furthermore, it would be clarifying for the field to use more precise terminology distinguishing G protein coupling (i.e. receptor binding), activation (G_α_–G_βγ_ dissociation), signaling (further downstream) and kinetics (e.g. activation rates).

Levering the new coupling datasets, we analyzed to what extent GPCR’s primary transducers differ across datasets and parameters (constitutive activity, log(E_max_/EC_50_) and activation rate (s^−1^)). We find that, of 18 GPCRs with couplings from constitutive activity, 17 (94%) have the same primary transducer G protein after agonist activation (Figure 4B). The primary G protein family is the same for 54 (68%) of 79 GPCR – primary transducer family pairs reported for 66 shared GPCRs in the GtoPdb, Bouvier GEMTA and Martemyanov FreeGβγ-Nluc datasets (Figure 4C left). The GtoPdb and Bouvier GEMTA datasets – focusing on E_max_ and EC_50_ – have a larger agreement, 85% with each other than to the kinetics-based dataset, (Martemyanov FreeGβγ-Nluc) (77% and 71%, respectively). This may indicate that, like for ligands, potency/efficacy metrics and kinetic parameters cannot be assumed to correlate. Furthermore, the Bouvier GEMTA and Martemyanov FreeGβγ-Nluc datasets show a marked lower agreement, 22% on the subtype-level (Figure 4C, right). Next, we investigated the G protein family profiles activated by the 66 shared GPCRs (79 combinations) in the GtoPdb, Bouvier GEMTA and Martemyanov FreeGβγ-Nluc datasets (Figure 4D). Here, only 0–2 couplings are unique to the kinetic assay, Martemyanov FreeGβγ-Nluc. Noticeably, 26%, 42% and 39% of Gi/o, Gq/11 and G12/13 family couplings are lacking only in GtoPdb and therefore represent new couplings supported by both other datasets. Of note, 54% of G_12/13_ couplings—which are underrepresented in GtoPdb (6)—are unique to the Bouvier GEMTA dataset. As new coupling datasets emerge, future studies should further advance our understanding of how GPCR – G protein selectivity (profiles, rank orders and primary transducers) differs across biosensors and parameters—as well as across cells/tissues with different effector stoichiometries/concentrations.

The first publication of GproteinDb contained homology models of 3081 GPCR–G protein complexes built using MODELLER (26). At the time, we were missing templates for, and could not model, several GPCR class/G protein family complexes. No homology models could be built of the G protein family G_12/13_ or class T GPCRs, and all and class B2 (Adhesion GPCRs) had to be modeled based on class B1 templates. Furthermore, models could not be built of class B1/G_q/11_, class B2/G_q/11_, class C/G_q/11_/G_s_, class F/G_q/11_/G_s_ complexes. This is addressed in this GproteinDb release that used many more templates and AlphaFold2-Multimer to attempt models of all theoretical complexes of the 16 human Gα proteins with 425 human GPCRs in GPCRdb. So far, this represents 5595 out of 6800 theoretical human GPCR-Gα complexes and more models will be added as they pass our quality filters (see Methods). This is a near-doubling of the number of structure models greatly expanding the GPCR–G protein ‘couplome’ for which it is possible to formulate structure-based hypotheses across a plethora of studies involving e.g. molecular dynamics, molecular mechanistic, mutagenesis, kinetics and drug design.

We focused on the Gα subunit, as it has the vast majority of receptor interactions, and it was not possible to compute high-quality models including Gβ and Gγ for the complete interactome. However, we will revisit this possibility as the AlphaFold2-Multimer algorithm continues to refine. We choose to model all theoretical G protein–GPCR complexes, as it is difficult to definitely rule out specific complexes that cannot form. For example, a complex not observed in one experiment may form in another experiment if changing the cell line or ligand (system and ligand bias, respectively). To help users assess the experimental support of complexes, we provide GPCR–G protein coupling data in the *Structure models* browser and individual pages of structure models.

The new data and visualization of interface interactions ties into a stream of scientific studies investigating determinants of GPCR – G protein coupling selectivity or promiscuity (6,33,45,48–49). It provides the opportunity to resolve missing sidechains interactions (due to lacking density) by analyzing refined structure complexes or to modelled complexes. The visualization allows comparison of sequence (biflare plot in Figure 3A) and structure (3D view in Figure 3B) and dissection of unique and common interactions across receptors (matrix in Figure 3D). This may contribute to ongoing discussions in the field regarding to what extend GPCR–G protein selectivity is driven by differences in sequence (33), structure (50,51), dynamic location and duration of intermolecular contacts at the periphery of the interface (45), tissues/cells with different protein stoichiometry (19), transient intermediate-state complexes (52) or cellular localization (53). Likely several mechanisms act in concert. For example recent analysis indicates that the volume of the G protein cavity, along with intracellular loops 2–3 (49), largely influences primary transducer coupling whereas secondary transducers may bind in a noncanonical fashion not requiring the same structural features and may instead be more dependent on sequence and other factors (48).

## Data Availability

All data is available via the web (Section ‘GproteinDb’ in https://gproteindb.org) and GitHub (https://github.com/protwis/gpcrdb_data). Documentation is available at https://docs.gproteindb.org. All open-source code can be obtained from GitHub (https://github.com/protwis/protwis) under the permissive Apache 2.0 License (https://www.apache.org/licenses/LICENSE-2.0). The source code was deposited on Zenodo (https://zenodo.org/records/10174368) as well as the data files (https://zenodo.org/records/10168935).
